# Trauma, immigration, and sexual health among Latina women: Implications for maternal–child well‐being and reproductive justice

**DOI:** 10.1002/imhj.21805

**Published:** 2019-07-23

**Authors:** Lisa R. Fortuna, Carmen Rosa Noroña, Michelle V. Porche, Cathi Tillman, Pratima A. Patil, Ye Wang, Sheri Lapatin Markle, Margarita Alegría

**Affiliations:** ^1^ Boston Medical Center Department of Psychiatry Boston Massachusetts; ^2^ Boston Medical Center Child Witness to Violence Boston Massachusetts; ^3^ Boston University, Wheelock College of Education and Human Development Boston Massachusetts; ^4^ La Puerta Abierta Philadelphia Pennsylvania; ^5^ Massachusetts General Hospital Disparities Research Unit and Harvard Medical School Boston Massachusetts

**Keywords:** immigrant, infant mental health, Latina, reproductive justice, traumatic stress, justicia reproductiva, inmigrante, latina, estrés traumático, salud mental infantil, Justice reproductive, Immigrées, Latina, Stress traumatique, Santé Mentale du Nourrisson, Reproduktive Gerechtigkeit, Einwanderer, Lateinamerikanerin, Traumatischer Stress, Psychische Gesundheit von Säuglingen, 生殖の公平性, 移民, ラテン系の, 外傷性ストレス, 乳幼児精神保健, 生殖公義, 移民, 拉丁裔, 創傷壓力, 嬰兒心理健康, العدالة الإنجابية, مهاجر, لاتينو, الإجهاد الناتج عن الصدمة, صحة الطفل النفسية

## Abstract

Latina immigrant women are vulnerable to traumatic stress and sexual health disparities. Without autonomy over their reproductive health and related decision‐making, reproductive justice is elusive. We analyzed behavioral health data from 175 Latina immigrant participants (*M* age = 35; range = 18–64) of the International Latino Research Partnership (ILRP) study. We used descriptive and inferential statistics to compare immigrant mothers of minor children to those without, regarding their psychological and reproductive health, and correlates of past exposure to sexual trauma. Over one third (38%) of ILRP participants had minor children, and 58% had citizenship in their host country. The rate for sexual assault was 30 and 61%, respectively, for physical assault; these rates were similarly high for women with and without minor children. Women who reported sexual assault scored significantly higher for depression, posttraumatic stress disorder, and substance‐abuse screens. Odds of experiencing sexual assault was highest for women who experienced physical assault (odds ratio = 10.74), and for those from the Northern Triangle (odds ratio = 8.41). Subgroups of Latina migrant mothers are vulnerable to traumatic stress and related sexual and mental health risks. Given these findings, we frame the implications in a reproductive justice framework and consider consequences for caregiver–child well‐being.

## RESUMEN

**Trasfondo**: Las mujeres latinas inmigrantes son vulnerables al estrés traumático y a las disparidades de salud sexual. Sin autonomía sobre su salud reproductiva y las decisiones que se deben tomar al respecto, la justicia reproductiva es difícil de alcanzar. **Métodos**: Analizamos información sobre las actitudes con respecto a la salud de parte de 175 inmigrantes latinas participantes (edad promedio 35; entre 18 y 64) del estudio de Investigación Conjunta Internacional de Asuntos Latinos (ILRP). Usamos estadísticas descriptivas y deductivas para comparar las madres inmigrantes de niños menores con aquellas sin ellos, sin tomar en cuenta su salud sicológica y reproductiva, y correlacionar el haber estado expuestas a trauma sexual en el pasado. **Resultados**: Más de un tercio (38%) de las participantes del grupo de ILRP tenían niños menores, y 58% tenían ciudadanía en el país donde residían. El promedio en cuanto a la agresión sexual fue de 30% y 61% en el caso de agresión física; estos promedios fueron similarmente altos tanto para mujeres con niños pequeños como mujeres sin niños pequeños. Las mujeres que reportaron agresión sexual tuvieron puntajes significativamente más altos en el caso de depresión, trastorno por estrés postraumático (PTSD) y exámenes de detección de abuso de sustancias. Las posibilidades de experimentar agresión sexual fue lo más alto para mujeres que experimentaron agresión física (OR = 10.74), y para aquellas del Triángulo del Norte (OR = 8.41). **Conclusiones**: Los subgrupos de madres latinas inmigrantes son vulnerables al estrés traumático y los relacionados riesgos de salud sexual y mental. Dados estos resultados, colocamos las implicaciones dentro de un marco de trabajo de justicia reproductiva y consideramos las consecuencias para el bienestar de quien le presta cuidados al niño.

## RÉSUMÉ

**Contexte** Les femmes immigrées latinas sont vulnérables au stress traumatique et aux disparités de santé sexuelle. Sans autonomie quant à leur santé reproductive et les décisions qui y sont liées, leur justice reproductive est insaisissable. **Méthodes** Nous avons analysé des données de santé comportementale de 175 participantes immigrées (moyenne d’âge 35 ans; éventail de 18 à 64 ans) de l’étude du partenariat de recherche international International Latino Research Partnership (ILRP). Nous avons utilisé des statistiques descriptives et déductives pour comparer les mères immigrées d'enfants mineurs à celles sans enfants, pour ce qui concerne leur santé psychologique et reproductive, ainsi que les corrélats d'exposition à un trauma sexuel dans le passé. **Résultats** Plus d'un tiers (38%) des participantes ILRP avaient des enfants mineurs et 58% détenaient la citoyenneté dans leur pays d'accueil. Le taux de violences sexuelles était de 30% et de 61% pour les aggressions physiques. Ces taux étaient aussi élevés chez les femmes avec ou sans enfants mineurs. Les femmes ayant déclaré des violences sexuelles ont fait état de scores bien plus élevés pour la dépression, le TSPT et la toxicomanie. Les probabilités de faire face à des violences sexuelles étaient les plus élevées chez les femmes ayant vécu une aggression physique (OR = 10,74), et pour celles du Triangle du Nord de l'Amérique centrale (OR = 8,41). **Conclusions** Des sous‐groupes de mères migrantes latinas sont vulnérables au stress traumatique et à des risques de santé mentale qui y sont liés. Au vu de ces résultats, nous encadrons les implications dans une structure de justice de reproduction et considérons les conséquences pour le bien‐être mère‐enfant.

## ZUSAMMENFASSUNG

**Hintergrund**: Lateinamerikanische Immigrantinnen sind vulnerabel für traumatischen Stress und Ungleichheiten in der sexuellen Gesundheit. Ohne Autonomie über ihre reproduktive Gesundheit und die damit verbundenen Entscheidungen ist reproduktive Gerechtigkeit schwer erreichbar. **Methoden**: Wir analysierten verhaltensbezogene Gesundheitsdaten von 175 lateinamerikanischen Immigrantinnen (Durchschnittsalter 35 Jahre; Range: 18–64 Jahre) aus der Studie der „International Latino Research Partnership“ (ILRP). Wir verwendeten deskriptive und inferentielle Statistiken, um immigrierte Mütter von minderjährigen Kindern mit solchen ohne minderjährige Kinder in Bezug auf ihre psychologische und reproduktive Gesundheit und hinsichtlich Korrelaten mit einer früheren Exposition gegenüber sexuellen Traumata zu vergleichen. **Ergebnisse**: Über ein Drittel (38%) der ILRP‐Teilnehmerinnen hatte minderjährige Kinder, und 58% hatten die Staatsbürgerschaft in ihrem Gastland. Die Rate für sexuelle Übergriffe betrug 30% und für körperliche Übergriffe 61%; diese Raten waren für Frauen mit und ohne minderjährige Kinder ähnlich hoch. Frauen, die über sexuelle Übergriffe berichteten, erzielten signifikant höhere Ergebnisse in Bezug auf Depression, PTBS und Drogenmissbrauch. Die Wahrscheinlichkeit, sexuelle Gewalt zu erfahren, war bei Frauen, die körperlichen Übergriffen ausgesetzt waren (OR = 10.74) und bei Frauen aus dem nördlichen Dreieck (OR = 8.41) am höchsten. **Schlussfolgerungen**: Subgruppen von immigrierten, lateinamerikanischen Müttern sind vulnerabel für traumatischen Stress und damit verbundene Risiken für die sexuelle und psychische Gesundheit. Vor dem Hintergrund dieser Ergebnisse setzen wir die Implikationen in den Rahmen der reproduktiven Gerechtigkeit und betrachten die Folgen für das Wohlbefinden der Bezugsperson und des Kindes.

## 抄録

ラテン系女性のトラウマ、移民、性の健康: 母子福祉と生殖の公平性の意味

**背景　**ラテン系移民女性は、外傷性ストレスや性の健康の相違に対して脆弱である。彼らの生殖の健康やそれに関連する意思決定についての自由がなくては、 生殖の公平性は、達成困難である。**方法　**我々は、「国際ラテン系研究パートナーシップ」 (ILRP) の研究における 175 人のラテン系移民の参加者(平均年齢 35 歳、範囲 18 歳～ 64歳)の行動に現れる精神保健のデータを分析した。我々は、未成年の子どもをもつ母親移民と未成年の子どもをもたない母親移民とを、心理的及び生殖の健康において、また、過去に晒された性的なトラウマとの関係にあるものについて比較するために、事実による統計と推定による統計を使用した。**結果　**ILRP 参加者の 3 分の 1 以上 (38％) は未成年の子どもがいて、 58％ は受け入れ国において市民権があった。性的暴行の割合は 30％で、身体的暴力は 61％ であった。これらの割合は、未成年の子どもがいようがいなかろうが、どちらの女性とも同様に高かった。性的暴行を報告した女性は、鬱病、 PTSD、物質乱用の評価が著しく高かった。性的暴行を体験した確率は、身体的暴力を体験した女性に最も高く(OR = 10.74)、そして、グアテマラ、ホンジュラス、エルサルバドルからの女性が最も高かった (OR = 8.41)。**結論　** 小集団のラテン系の移民女性は、外傷性ストレスやそれに関連した性的な健康や精神保健のリスクに対して脆弱である。これらの調査結果より、我々は、生殖の公平性の枠組みにおける繋がりを形作り、養育者−子どもの福祉に関する結果を考察する。

## 摘要

**拉丁裔婦女的創傷丶移民和性健康:對母嬰健康和生殖公義的意義**

**背景**拉丁裔移民婦女容易受到創傷性壓力和性健康差異的影響。如果沒有生殖健康和相關決策的自主權, 生殖公義就難以實現。**方法**我們分析國際拉丁裔研究夥伴 (ILRP) 的 175 名拉丁裔移民參與者 (平均年齡 35 歲;範圍 18–64 歲) 的行為健康數據。我們使用描述性和推論性統計數據, 來比較未成年子女的移民母親與沒有子女的移民母親, 在心理和生殖健康, 以及過往性創傷的相關因素。**結果**超過三分之一 (38％) 的 ILRP 參與者有未成年子女, 58％ 的人在其所在國家擁有公民身份。性侵犯**和**身體侵犯的百分率分別為 30％和 61％; 對於有和沒有未成年子女的婦女, 這些比率同樣很高。報告性侵犯的女性在抑鬱丶創傷後應激障礙和藥物濫用篩查方面得分顯著較高。被侵犯身體的女性 (OR = 10.74) 和來自北三角的女性 (OR = 8.41) 經歷性侵犯的機率最高。**結論**一些拉丁裔移民母親易受創傷性壓力和相關的性和心理健康風險。我們在生殖公義框架中討論這些發現的含義, 並考慮其對照顧者 – 兒童福祉的影響。

## ملخص

الصدمة والهجرة والصحة الجنسية بين سيدات اللاتينو: تطبيقات رفاهية الأمهات والأطفال والعدالة الإنجابية

نساء اللاتينو المهاجرات معرضات للإجهاد العصبي الناتج عن الصدمة النفسية وتفاوتات في الصحة الجنسية. فبدون سيطرتهم الكاملة على صحتهم الإنجابية واتخاذ القرارات ذات الصلة، تصبح العدالة الإنجابية غير متحققة بشكل واضح. **الدراسة **: قمنا بتحليل البيانات الصحية السلوكية من 175 مشاركا من المهاجرين اللاتينو (متوسط العمر 35 سنة؛ متراوحا بين 18–64) من ضمن بيانات دراسة الشراكة الدولية للبحوث اللاتينية (ILRP). واستخدمنا إحصاءات وصفية واستنتاجية لمقارنة الأمهات المهاجرات من الأطفال القصر بغيرهن، فيما يتعلق بصحتهن النفسية والإنجابية، ومعاملات ارتباط تعرضهن في الماضي للصدمات الجنسية. **النتائج **: أكثر من الثلث (38%) من المشاركين في دراسة “ILRP” كان لديهم أطفال قصر، وكان 58 في المائة منهم حاصلين على الجنسية في البلد التي هاجروا إليها. وكان معدل الاعتداء الجنسي 30 في المائة و 61 في المائة بالنسبة للاعتداء البدني؛ وكانت هذه المعدلات مرتفعة بالمثل بالنسبة للنساء سواء كان عندهن أطفال قصر أو لا . وسجلت النساء اللاتي أبلغن عن اعتداء جنسي معدلات أعلى بكثير من حالات الاكتئاب واضطراب ما بعد الصدمة واختبارات إساءة استعمال المخدرات. وكانت احتمالات التعرض للاعتداء الجنسي هي الأعلى بالنسبة للنساء اللاتي تعرضن للاعتداء البدني (معامل احتمال = 10.74)، وبالنسبة للنساء من المثلث الشمالي (معامل احتمال = 8.41). **الاستنتاجات **: مجموعات فرعية من أمهات اللاتينو المهاجرات عرضة للإجهاد العصبي وما يتصل بذلك من مخاطر الصحة الجنسية والنفسية. وفي ضوء هذه النتائج، نصوغ التطبيقات في إطار العدالة الإنجابية وننظر في العواقب على رفاهية مقدمي الرعاية والطفل.

## 1 INTRODUCTION

Reproductive justice entails women and girls having autonomy over decisions affecting their own well‐being and that of their families and communities (Smith, 2005). Coined first by African American women in a 1994 convention on reproductive health, the term *reproductive justice* conceptualizes the combining of reproductive rights and social justice, and is a repudiation of a centuries‐old history of practices meant to limit reproductive options for women of color, indigenous people, immigrants, and other marginalized communities (Copelon & Petchesky, 1995). The critical values of reproductive justice are “(1) the right *not to have* a child; (2) the right to *have* a child; and (3) the right to *parent* children in safe and healthy environments” (Ross & Salinger, 2017, p. 65). The framing of these issues from a reproductive justice lens readjusts our focus onto public policies that have an economic and health impact, particularly on vulnerable women, and that are rarely explored in the traditional debates on abortion, reproductive choice, and caregiver well‐being. This current study examines factors that may diminish reproductive justice for Latina immigrants with children and how this might impact their well‐being.

### 1.1 Immigrant women and reproductive justice

Marginalized women of color experience systematic barriers for accessing contraception, comprehensive sex education, sexually transmitted infection prevention and care, alternative birth options, adequate prenatal and pregnancy care, domestic violence assistance, adequate wages to support their families, safe homes, healthcare, mental health services, and more. For Latina immigrant and refugee women, policies that influence migration and mental health/healthcare access and address poverty are of vital importance for reproductive justice, maternal health, and caregiver–child well‐being. The National Women's Law Center ( 2017) highlighted that Latina immigrant women seeking to escape sexual trauma and community violence are stereotyped as opportunistic; as simply seeking to give birth and gain employment in the United States. Such characterizations have been used to justify the codification of immigration policies that effectively limit access to healthcare and foster discrimination and fear (National Women's Law Center, 2017). In fact, some federal health insurance policies directly prevent access to seek reproductive health services, preventive care, or other treatment for immigrant and refugee women and their families (National Women's Law Center, 2017).

### 1.2 Immigrant women's experiences of violence, trauma, and marginalization

Experiences of trauma, violence, and poor mental health complicate the circumstances and barriers for attaining reproductive justice among Latina immigrant women. They may be at increased risk for intimate partner violence (IPV), as compared to other minority groups, placing them at greater risk for posttraumatic stress disorder (PTSD), lifelong health problems, and disparities (Galano, Grogan‐Kaylor, Stein, Clark, & Graham‐Bermann, 2016). Kaltman, Green, Mete, Shara, and Miranda (2010) found that Latina immigrant women who had experienced IPV from rape and physical violence were at highest risk for PTSD and depression comorbidity. Because IPV can compromise the caregivers’ ability for attunement and empathy with their child, it can also decrease their capacity of appraising and responding to danger in the environment. Thus, these become vulnerabilities for caregiver–infant well‐being (Lieberman & Van Horn, 1998; Lieberman, Van Horn, & Ippen, 2005) and can lead to impaired parenting practices. IPV can disrupt the caregiver–child relationship and is especially toxic for young children who are exposed to violence, as it damages the perception of the caregiver (mother) as a reliable and safe protector (Lieberman & Van Horn, 2008).

In addition, disenfranchised Latina immigrant mothers face the consequences of gender, class, racial oppression, and consequently poverty, resulting in poor access to protective systems of social support and mental health services which could otherwise mitigate developing posttraumatic stress and revictimization (Crenshaw, 2005; Galano et al., 2016). Undocumented Latina immigrants are more likely to have higher rates of IPV and involvement with the child welfare system, as compared to women who are permanent residents or citizens (Ogbonnaya, Finno‐Velasquez, & Kohl, 2015). Risk is highest for IPV during pregnancy and after childbirth, and immigrant status can increase dependency on a partner, leaving women less likely to report abuse and to seek asylum or legal aid (Coutinho et al., 2015) out of fear of deportation and family separation as well as stigma.

### 1.3 Healthcare policy, reproductive health, and mental health services

Health reform initiatives actively bar immigrant women from receiving safety net health benefits (National Latina Institute for Reproductive Health, n.d.). While the Patient Protection and Affordable Care Act (2010) expanded coverage for preventive healthcare such as family planning and screening for sexually transmitted diseases and domestic violence, it excluded undocumented immigrants from these benefits. Other policies legislated within the last 10 years have restricted access to abortion services in community clinics and caused reductions in access to affordable reproductive health services and contraception care (Nash, Gold, Ansari‐Thomas, Cappello, & Mohammed, 2017). These laws disproportionately impact undocumented women, but also other low‐income women who rely more heavily on community clinics to receive reproductive health services.

In the United States, there is a multiple‐payer system (Medicaid, Medicare, private insurance, and safety net programs) with great diversity in access, the copayments billed to the patient, and in which many insurance plans limit the total number of mental health visits (Saloner, Bandara, Bachhuber, & Barry, 2017). Undocumented immigrants and authorized immigrants with less than 5 years of legal residence cannot receive federal insurance subsidies or enroll in Medicaid (a social healthcare program for low‐income individuals). Most Latina immigrants, even those who have documented status, receive mental healthcare in the United States through community‐based clinics where a primary care doctor treats them or refers them if they accept public insurance or the clinic receives federal subsidies; but they still confront colossal barriers in the process of care (Agency for Healthcare Research and Quality, 2015). Spain has a single‐payer system in which copayments for mental healthcare visits are not common and an unlimited number of mental health visits are allowed (Dezetter et al., 2013). In both countries, the main entrance to the mental healthcare system is through primary care centers, and significant barriers to specialty mental healthcare exist (Falgas et al., 2017). These policies, whether part of immigration, social welfare, or healthcare systems, determine what services and health‐promoting resources immigrant women can access for themselves and their children.

## 2 RELATIONSHIP OF MATERNAL WELL‐BEING AND REPRODUCTIVE JUSTICE

Maternal well‐being and reproductive justice are interrelated and have direct implications for the development of an infant's capacity for emotional connection, regulation, and expression, as influenced by the quality of the child–caregiver relationship (Balbernie, 2002). Mothers in a deeply traumatized state have more difficulty performing the basic tasks of attunement and mirroring necessary for healthy attachment with their child, who thus experiences relational trauma (Schore, 2013). Greater understanding of these mechanisms along with increased screening for Latino immigrant families, especially pregnant and new mothers and their infants, are essential to mitigate the effects of transgenerational trauma (Phipps & Degges‐White, 2014). Traumatic stress, violence exposure, and associations with reproductive justice, policy, and healthcare have been understudied.

**Figure 1:**
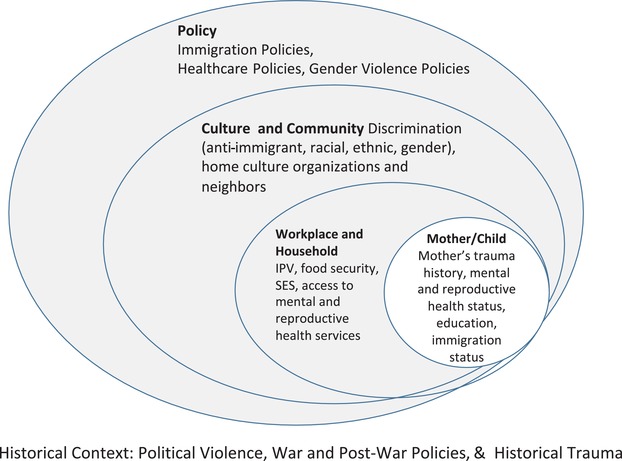
Ecological model of caregiver–child well‐being

In this article, we use an ecological model (Bronfenbrenner, 1979) to represent the multiple distal and proximal risk and protective factors which determine the quality of a caregiver–infant relationship and the multifactorial factors that impact these families. We examine the risks to these relationships, which we frame within a larger sociopolitical context (see Figure 1). Latina immigrant women are particularly vulnerable to trauma and sexual health disparities, yet there has been limited research describing the implications of these experiences for their psychological and reproductive health or for maternal–child well‐being. The ecological model guided our approach in selection of measures that investigate immigrant women's individual characteristics and immigrant status within relational contexts and community in two separate countries that historically have had large influxes of immigrants, even as policies vary. We use a combination of approaches to understand the reproductive health of immigrant Latinas. Our empirical analysis of study data best captures individual‐level variables that are associated with risk for IPV, sexual trauma, and maternal mental health problems among Latina immigrant mothers. It is supplemented by a composite case vignette from our research and clinical experience that augments our more limited measures of the exo‐ and macrosystems.

This article reports on findings from a clinical research study of Latino immigrants in the United States and Spain (International Latino Research Partnership; Alegría et al., 2019), which is unique in its investigation of trauma experiences and psychological outcomes in the context of global migration (Ramos et al., 2017). We use survey methods to examine factors that have an impact on Latina immigrant women's reproductive and emotional health, including the prevalence of exposure to interpersonal and sexual violence. We evaluate whether there are differential risk factors for mothers of minor children, as compared to women without children under the age of 18, among Latina immigrant mothers. What are the variations in risk factors, if any? We identify factors associated with sexual trauma and mental health, and specific recommendations for intervention, prevention, and policy efforts using an illustrative vignette that reflects our ecological model and is a composite from our clinical experience, written to protect the privacy of any one individual. We frame results in a reproductive justice framework by considering caregiver–child well‐being, reproductive health, and implications for clinical practice and policy for supporting Latina immigrant women and families.

## 3 METHODS

Descriptive and inferential statistics were conducted using quantitative data derived from a mental health and substance‐abuse screening interview and baseline data collected from study participants between September 2014 and May 2016 as part of the International Latino Research Partnership study (ILRP; Alegría et al., 2019) funded by the National Institute on Drug Abuse. A primary focus of the ILRP was to unite research institutions and community clinics in the United States and Spain to conduct cross‐national comparative research investigating Latino migrants’ behavioral health service needs. The ILRP study included behavioral health services research focused on rapid screening and referral for 341 men and women in the United States and Spain as well as testing the feasibility, acceptability, and efficacy of integrated behavioral health services in primary care clinics and community‐based organizations including a transdiagnostic therapy intervention for anxiety, depression, PTSD, mild to moderate substance‐use problems, and HIV prevention for migrant Latinos (Fortuna, Ramos, Fuentes, Falgas, & Alegría, 2017). For this analysis, a subset of the 175 women were included; the remaining 166 male participants were excluded. ILRP participants were recruited in waiting rooms of primary care clinics and community agencies in Boston, Massachusetts, and Madrid and Barcelona, Spain. Local Spanish‐speaking research staff in each participating site obtained informed consent from participants and conducted a 90‐min, audio‐recorded interview that included demographic questions and a battery of measures validated in English and Spanish (discussed later). All research procedures were approved by the Human Subjects Internal Review Boards of each of the participating research institutions.

### 3.1 Sample

The study sample was recruited from primary care clinics and community‐based organizations that offer social services to immigrant populations in the United States and Spain. Both countries receive Latino immigrants; however, the pathways differ as most immigrants to the United States come from Central America and the Caribbean. Spain has the second‐largest Latino immigrant population, with pople from Argentina, Bolivia, Chile, Colombia, Costa Rica, Cuba, Dominican Republic, Ecuador, El Salvador, Guatemala, Honduras, Mexico, Nicaragua, Panama, Paraguay, Puerto Rico, Uruguay, and Venezuela. There are key differences upon arrival for immigrants in Spain versus those in the United States. First, Spanish is the official spoken language in Spain. However, cultural differences between Latin Americans and Europeans exist, unemployment and underemployment rates are highest among immigrant populations in Spain, and even while pathways to citizenship are supported, there remains significant anti‐immigrant sentiment (Batsaikhan, Darvas, & Raposo, 2018).

Inclusion criteria included positive screening for elevated mental health symptoms and/or substance‐use problems (descriptive statistics for the short version of the Alcohol Use Disorders Identification Test (AUDIT; Babor, Higgins‐Biddle, Saunders, & Monteiro, 2001), the Drug Abuse Screening Test (DAST‐10; Skinner, 1982), and a selection of questions from the Benzodiazepine Dependence Questionnaire (BDEPQ; Minaya, Fresan, Cortes‐Lopez, Nanni, & Ugalde, 2011) are provided in the Appendix, but are beyond the scope of this analysis). Participants completed a baseline assessment for the ILRP intervention study (*n* = 175 women: 44 in Boston, 49 in Madrid, 82 in Barcelona; males excluded from this analysis). There were a higher proportion of participants from Spain, where there were two sites compared to one in the United States, with the highest recruitment in Barcelona. Experiences for women in the two contexts have shown similar outcomes (Collazos et al., 2019; Ramos et al., 2017). Most women were first‐generation immigrants (born in a country other than the interview site), comprising 99% of the Spain sample and 82% of the Massachusetts sample. Boston participants were born in Central American countries (18% El Salvador, 11% Guatemala), second‐generation immigrant or migrant United States (18%), Puerto Rico (20%), Dominican Republic (18%), Colombia (9%), and Mexico (5%); no participants were born in Spain. Most participant women in Spain were of South American origin (Argentina 2%, Bolivia 14%, Brazil 3%, Chile 4%, Colombia 7%, Ecuador 22%, Paraguay 4%, Peru 14%, Venezuela 19%), with only 6% from Central American countries (El Salvador, Guatemala, or Honduras), and fewer from Mexico (5%), Cuba (>1%), and Spain (>1%). Women in Barcelona were younger, migrated at a younger age, and had a lower education level, on average, than did women in Madrid or Boston. An important difference is that Spain has universal healthcare coverage that includes provisions for immigrants. Immigrants from Central America may have combined economic and refuge‐seeking reasons for migration. There were no site differences on trauma‐exposure measures (described later). The percentage of participants speaking Spanish as their primary language was 93% in Boston, 96% in Madrid, and 94% in Barcelona. On average, women had lived 10 years (*SD* = 8.25) in their resident country; however, time spent in the United States for immigrant Latinas was longer (*M* = 16.3, *SD* = 11.2), range = 1 to 43 years, as compared to those in Spain (*M* = 8.7, *SD* = 6.5), range = <1 to 40 years.

### 3.2 Measures

#### 3.2.1 Maternal status and sociodemographics

Women were categorized as having minor‐age children if they reported having one or more children under the age of 18 years. Some of these women may also have adult children, but are still categorized in this group if they have at least one child under 18. Self‐report sociodemographic information included current age, country of origin, country of residence, citizenship status, race, language, education level, number of children ages 17 and under, and family economic status (i.e., live very well or comfortably; live check‐to‐check or poor), highest level of education (i.e., less than high school; completed high school/GED, or vocational school), and having or not having a primary sexual partner.

#### 3.2.2 Mental health and sexual health profile

All measures included in this article have been validated and/or used extensively with Latinas who are Spanish speakers, in addition to English‐speaking populations. This includes the AUDIT (Babor et al. 2001), which has been developed, validated, and used by the World Health Organization in multiple languages and cultural contexts. Other measures were used in the NLAAS, the largest epidemiological study of Latinos in the contiguous mainland United States. The NLAAS included a focus on testing measures for *cultural relevance* by formulating the research problem with attention to the fundamental cultural and contextual differences of Latinos and Asians, as compared to the mainstream population (Alegría, Vila et al., 2004). Reported psychometrics were calculated from the full sample of ILRP study participants. Depression symptoms were measured using the 9‐item Patient Health Questionnaire (PHQ‐9; Huang, Chung, Kroenke, Delucchi, & Spitzer, 2006; Kroenke, Spitzer, & Williams, 2001), Cronbach's α = .85. Anxiety symptoms were measured with the General Anxiety Disorder screener (GAD‐7; Spitzer, Kroenke, Williams, & Löwe, 2006), Cronbach's α = .86. PTSD symptoms were measured using the Post‐Traumatic Stress Disorder Checklist, Civilian version (PCL‐5; Blanchard, Jones‐Alexander, Buckley, & Forneris, 1996), a self‐report measure of symptoms of PTSD according to the *Diagnostic and Statistical Manual of Mental Disorders (DSM‐5)* (American Psychiatric Association, 2013), Cronbach's α = .94.

#### 3.2.3 Sexual risk behaviors

We categorized the sexual partner status in the last 12 months into four categories: only having a main partner, only having a casual partner, having both a main and a casual partner, and having neither a main or a casual partner. Given the sexual partner status in the last year, we asked participants about the number of sexual experiences and whether a male and/or female condom was used during their sexual encounter. Finally, we categorized sexual behavior status during the last 12 months into three categories: unprotected sexual behavior if ever engaged in sex without a condom during the last 12 months, protected sexual behavior if condom was used all the time, and no sexual behavior if no sexual partners were reported for the last 12 months.

#### 3.2.4 Traumatic stress exposure and symptoms

Trauma exposure was assessed with the Brief Trauma Questionnaire (BTQ; Schnurr, Vielhauer, Weathers, & Findler, 1999), a 10‐item self‐report measure that includes participant perception of risk of death/serious injury and constructed as a binary variable indicating whether the respondent had experienced any traumatic event. This 10‐item checklist of traumatic events administered by trained interviewers was used to assess physical assault and sexual trauma exposure. In addition to questions about specific events, participants were further asked about whether they felt their lives were in danger or if they had experienced serious injuries because of that trauma. Exposure to a specific trauma event was coded as 1 if women indicated that they experienced the event and it resulted in serious injuries and/or felt life‐threatening; otherwise, it was coded as 0. Physical assault was constructed using two items from the checklist related to physical abuse: (a) Before age 18 were you physically punished or beaten by a parent or caregiver to the point of injury, and (b) Were you ever attacked, beaten, or mugged by someone other than a parent or caretaker, such as a stranger, friend, or family member (which could indicate interpersonal violence). Physical assault was coded as 1 if either of the items was endorsed, and 0 if neither was endorsed. Sexual trauma exposure was measured using a single question asking whether they had ever been forced or pressured to have unwanted sexual contact, coded as 1 if *Yes* and 0 if *No*.

#### 3.2.5 Cultural, contextual, and psychosocial factors

We included measures used in the NLAAS (Alegría, Takeuchi et al., 2004) that evaluate experiences of discrimination, sense of belonging, and the immigration experience. The three‐item Discrimination scale assessed whether these experiences were due to being Latino or not speaking English, Cronbach's α = .71. The immigration experience was assessed via participant report of citizenship status in the country of residence, age of immigration, and number of years lived in the host country. These questions were derived from the Mexican American Prevalence and Services Survey (Vega, Kolody, Valle, & Hough, 1986). The six‐item Acculturation Stress Scale measures the level of stress in the adaption to the new culture, range = 0–18, with a higher score indicating a higher level of stress due to acculturation, Cronbach's α = .70.

### 3.3 Composite vignette

The authors each have at least 15 to 20 years of experience in serving immigrant populations and/or conducting research with Latino immigrants (Alegría, Álvarez, & DiMarzio, 2017; Alegría, NeMoyer, Falgas Bague, Wang, & Alvarez, 2018). All authors reviewed the vignette for face validity and accurate representation of clinical issues for Latina immigrants. Vignettes are short stories about a hypothetical person, traditionally used within research (quantitative or qualitative) to gather participant perspective on sensitive topics (Gourlay et al., 2014). However, in this case, the vignette serves to contextualize our quantitative results and to highlight in our discussion section critical issues related to reproductive justice, specifically the association of traumatic stress and Latina immigrant mental health, on reproductive health and caregiver–infant well‐being. (See vignette in section 5.1: Illustrative clinical example.)

### 3.4 Analyses

Given the small frequency of missing data (3% or less in most variables), the analytic sample includes only the sample with nonmissing data in all reported variables used in the analyses. Descriptive and Fisher's exact statistics were used to compare women with and without minor children (ages 0–17 years) on psychosocial, psychological, and health measures. We conducted both Fisher's exact and chi‐square tests, and report on Fisher's for this article as it is a comparatively more conservative approach with small sample sizes or highly unequal cell distribution. Building on these descriptive analyses, logistic regression models were fit to test for correlates of sexual trauma history, beginning with maternal status, exposure to sexual risk, migration information and demographics, discrimination and acculturative stress, and psychological measures. To address the problem of collinearity between psychosocial, psychological, and health measures, we used a backward elimination approach to remove predictors whose association was not statistically significant, *p* > 0.25. The results of final models were presented as odds ratios (OR) with 95% confidence intervals using Stata: Release 14 (StataCorp, 2015).

## 4 RESULTS

Over one third of the sample (36%) included mothers of minor children younger than 18 years of age (Appendix Table A1). Women with minor children tended to be older adults (age range = 35–49) whereas women without minor children tended to be younger (ages 18–34). Women with minor children were also more likely to currently be citizens in the country of residence, to have spent more years in the host country, and to have lower education levels, as compared to women with no minor children.

Almost 30% (28.6%) of women reported exposure to sexual trauma (Table 1). Women from the Northern Triangle were more likely to have experienced sexual trauma, as compared to their counterparts from other regions. Report of sexual trauma was highly associated with having reported physical assault. Screening positive for mental health disorders (increased likelihood of elevated symptoms of depression, generalized anxiety, PTSD, and drug abuse including benzodiazepines) were women who had experienced sexual trauma. They also had been in the host country for a longer time and scored higher on the acculturation stress measure.

**Table 1:** Sample descriptive summary by sexual trauma status (*N* = 154)

	Total (*N* = 154)		Women without sexual trauma exposure (*n* = 110)		Women with sexual trauma exposure (*n* = 44)		
	*n*	%	*n*	%	*n*	%	*P*‐value
Age category							
18–34	79	51.3	62	56.4	17	38.6	0.062
35–49	49	31.8	29	26.4	20	45.5	
50+	26	16.9	19	17.3	7	15.9	
Site							
Boston	30	19.5	20	18.2	10	22.7	0.159
Madrid	46	29.9	29	26.4	17	38.6	
Barcelona	78	50.6	61	55.5	17	38.6	
Northern Triangle							
Not from El Salvador, Guatemala, or Honduras	137	89.0	103	93.6	34	77.3	0.003
From El Salvador, Guatemala, or Honduras	17	11.0	7	6.4	10	22.7	
U.S/Spain citizenship							
Noncitizen	67	43.5	52	47.3	15	34.1	0.136
Citizen	87	56.5	58	52.7	29	65.9	
Race							
White	27	17.5	20	18.2	7	15.9	0.282
Black	7	4.5	4	3.6	3	6.8	
Indigenous/Native American	14	9.1	9	8.2	5	11.4	
Hispanic/Latino/Caribbean	9	5.8	4	3.6	5	11.4	
Mixed	97	63.0	73	66.4	24	54.5	
Education Level							
<High school	52	33.8	40	36.4	12	27.3	0.281
High‐school diploma, GED, Vocational school, or more	102	66.2	70	63.6	32	72.7	
Last year sexual partner status							
Only have main partner	71	46.1	54	49.1	17	38.6	0.441
Only have casual partner	16	10.4	9	8.2	7	15.9	
Both main and casual partner	35	22.7	24	21.8	11	25.0	
Neither main nor casual partner	32	20.8	23	20.9	9	20.5	
No. of Children							
<3	149	96.8	108	98.2	41	93.2	0.114
≥3	5	3.2	2	1.8	3	6.8	
Physical Assault							
No	60	39.0	53	48.2	7	15.9	0.000
Yes	94	61.0	57	51.8	37	84.1	
Last year sexual behavior status							
Unprotected sexual behavior	84	54.5	55	50.0	29	65.9	0.119
Protected sexual behavior	37	24.0	31	28.2	6	13.6	
No sexual behavior	33	21.4	24	21.8	9	20.5	
Met criteria for PTSD (PCL‐5 33+)	69	44.8	40	36.4	29	65.9	0.001
Depression (PHQ‐9) (0–27)	12.9	5.7	12.0	5.8	15.0	5.1	0.003
Generalized anxiety (GAD‐7) (0–21)	10.0	4.9	9.2	4.7	11.8	4.9	0.003
PTSD (PCL) (0–80)	30.0	17.1	26.0	16.1	40.0	15.3	0.000
Alcohol abuse (AUDIT) (0–12)	4.5	3.2	4.5	3.2	4.7	3.2	0.706
Drug abuse (DAST) (0–9)	0.9	1.8	0.8	1.7	1.4	2.0	0.045
Benzodiazepines (0–20)	3.3	5.1	2.8	4.6	4.7	5.9	0.032
Discrimination Scale (0–54)	17.6	7.5	17.3	7.2	18.2	8.1	0.486
Acculturative Stress Scale (0–18)	3.3	2.7	2.9	2.5	4.3	2.9	0.002
*M* age of immigration	25.4	9.7	25.3	10.3	25.7	8.2	0.835
Length of time in resident country	10.2	8.2	9.3	7.2	12.2	9.9	0.046

*Note*. Sample includes only participants with nonmissing data in all reported variables. Fisher's exact and *t* test were used to assess subgroup differences for categorical and continuous variables. GED = general equivalency diploma; PTSD = posttraumatic stress disorder; PCL‐5 = Post‐Traumatic Stress Disorder Checklist, Civilian version; PHQ‐9 = Patient Health Questionnaire (9‐item); GAD‐7 = General Anxiety Disorder screener; AUDIT = Alcohol Use Disorders Identification Test; DAST = Drug Abuse Screening Test.

Results from the logistic regression (Table 2) showed that the odds of sexual trauma were significantly lower among women who reported always engaging in protected sex or who reported no sexual behaviors, OR = 0.21, 95% CI [0.06, 0.73]. Women who reported experiences of physical assault had over 10 times the odds, OR = 12.08, 95% CI [3.69, 39.59], of also reporting experiences of sexual trauma. Women from the Northern Triangle had 8 times the odds, OR = 7.65 95% CI [1.90, 30.76], of sexual trauma, as compared to women not from this region. The odds of experiencing sexual trauma were higher for women with citizenship, OR = 2.98, 95% CI [1.02, 8.69], and who experienced greater acculturation stress, OR = 1.44, 95% CI [1.16, 1.80]. There were no statistically significant associations between sexual partnership status, race, education level, age at immigration, or everyday discrimination on the likelihood of reporting experiences of sexual trauma. Women with minor children had over twice the odds of reporting physical assault versus those without minor children, OR = 2.42, 95% CI [1.03, 5.69], and there was slightly higher increase of risk associated with older age at immigration, OR = 1.05, 95% CI [1.00, 1.09], and reporting experiences of discrimination, OR = 1.07, 95% CI [1.00, 1.13].

**Table 2:** Logistic regressions for associations between covariates and exposure to sexual trauma and physical assault (*N* = 154)

Characteristic	Sexual trauma exposure	Physical assault exposure
Women with children	0.51	2.42*
	[0.18, 1.39]	[1.03, 5.69]
Last year sexual behavior status (ref = Unprotected sexual behavior)		
Protected sexual behavior/no sexual behavior	0.21*	2.37
	[0.06, 0.73]	[0.94, 5.96]
Last year sexual partner status (ref = Have sexual partner)		
Do not have sexual partner	3.15	0.38
	[0.70, 14.20]	[0.12, 1.19]
Physical assault	12.08***	N/A
	[3.69, 39.59]	
Site (ref = Boston)		
Madrid	3.08	2.45
	[0.61, 15.50]	[0.67, 8.96]
Barcelona	1.47	1.93
	[0.30, 7.16]	[0.56, 6.70]
Northern Triangle (ref = not El Salvador, Guatemala, or Honduras)		
Country is El Salvador, Guatemala, or Honduras	7.65**	1.58
	[1.90, 30.76]	[0.44, 5.73]
Race (ref = White)		
Black	3.69	0.68
	[0.40, 34.25]	[0.10, 4.74]
Indigenous/Native American	1.60	0.59
	[0.24, 10.72]	[0.13, 2.67]
Hispanic/Latino/Caribbean	1.39	1.79
	[0.13, 15.43]	[0.25, 12.54]
Mixed	0.43	1.55
	[0.11, 1.72]	[0.57, 4.18]
Education level (ref = <High school)		
High‐sschool diploma, GED, Vocational school, or more	3.22*	0.76
	[1.07, 9.68]	[0.33, 1.72]
*M* Age of immigration	0.98	1.05*
	[0.94, 1.04]	[1.00, 1.09]
Citizenship	2.98*	1.20
	[1.02, 8.69]	[0.55, 2.61]
Discrimination Scale	0.93*	1.07*
	[0.87, 1.00]	[1.00, 1.13]
Acculturative Stress Scale	1.44**	1.00
	[1.16, 1.80]	[0.85, 1.19]
Pseudo *R* ^2^	0.304	0.121

*Note*. Odds ratios and 95% confidence intervals were reported. GED = general equivalency diploma.

^*^ *P* < .05. ^**^ *P* < .01. ^***^ *P* < .001.

## 5 DISCUSSION

Gender‐based and IPV, socioeconomic factors and policies and practices that limit protections from violence, are an important part of the reproductive justice discourse for many Latina immigrant women. Although immigrant women in our sample with and without minor‐age children had experienced substantial trauma and social challenges, we found a trend toward more anxiety in women with minor children. These women were less educated and more likely to have resided longer as immigrants in the United States or Spain, as compared to their counterparts without children under the age of 18 years. Experiences of violence can contribute to a higher prevalence of depression and anxiety in the population (Fortuna, Porche, & Alegría, 2008).

Our findings from the ILRP study demonstrate that exposure to trauma is common especially among subgroups of immigrant women from Latin America residing in the United States and Spain. We found a higher likelihood of reports of sexual trauma among women from Central America's the Northern Triangle, as compared to other Latina immigrant women in the study. Controlling for age, women who have lived for a longer time in the host country also show a relatively higher prevalence of exposure to sexual trauma, which may be related to being older and/or having a longer period of risk for exposure both pre‐ and postmigration. The prevalence of reported childhood sexual abuse in the three Central American countries of the Northern Triangle has been found to range from 5 to 8% (Gómez & Speizer, 2009). Women from Guatemala and El Salvador who have experienced childhood sexual abuse have increased likelihood of being in violent relationships, as compared to women who do not have these experiences (Arias, 2004; Gómez & Speizer, 2009).

Exposure to sexual trauma poses a sexual health risk. Our findings from the ILRP study suggest that women without a sexual trauma history are more likely to engage in protected sex with a condom, as compared to women with sexual trauma histories, again pointing to the potential saliency of this risk factor. The ILRP study included an intervention for the treatment of mental health and substance‐use problems as well as offering HIV risk prevention. Many women participating in the intervention study described fear that their partner would accuse them of mistrust or would assume she was not being monogamous if she tried to negotiate condom use. In some cases, this incited the potential risk of violence. Women shared that these unsafe situations limited their ability to reduce their risk for HIV and sexually transmitted infection and/or engage in regular HIV testing.

In our practice and research with immigrant Latinas, we have found that experiences of sexual violence and IPV frequently co‐occur and result in cumulative risk and barriers for healthcare as well as safety for the woman and her children. Many women endure separations from very young children, leaving their children with relatives in their country of origin before seeking a safe reunification later, sometimes many years later. Sexual abuse and sexual violence histories may also lead to stigmatization and isolation (Denov, 2015). Women may experience educational and economic challenges because of the cumulative effects of gender‐based violence and discrimination and the stress of parenting under circumstances of disadvantage and oppression, all which have a potentially negative impact on caregiver–infant well‐being.

### 5.1 Illustrative clinical example

Using the following vignette, which is a composite of our clinical experiences and research, we illustrate common themes relevant to our study findings and as faced by immigrant women and mothers, their circumstances, and how they try to overcome these risks for themselves and their children.

Luisa is a 16‐year‐old girl who recently arrived from El Salvador to be reunited with her mother after receiving death threats from a local gang in her community. Her mother had arranged for her to be smuggled through Mexico by a coyote smuggler with other migrants. Upon entering Mexico, she was separated from the group and found herself lost, although near the U.S. border. Gang members kidnapped her and held her in a room in an undisclosed building for several days, during which time she was raped along with several other young girls. She was released and, although sexually traumatized, made her way to the border, crossing into the hands of U.S. Immigration and Customs Enforcement. Upon being detained and then sent to a large city in the Northeast to live with her mother, she discovered she was 3 months pregnant, clearly because of the rape. Note that Luisa did not disclose the rape to anyone during the border crossing nor to her mother upon initial reunification.

At age 16, despite arriving to the United States without authorization, Luisa could not access adequate prenatal care in her new city. Without a social security number, she was not able to receive in‐school childcare and instead relied on her mother so that she could complete school. Luisa felt extremely traumatized by the birth of the child, as it was a not only a reminder of the rape but also of her own abandonment as an infant. Due to language and insurance barriers, this new parent was unable to access postpartum support outside of routine “well‐baby” visits within a local clinic. In addition, she has been socially isolated given her newcomer status, non‐English speaking skills, lack of access to adolescent programming for teen mothers, and lack of an established peer‐community support. Luisa faced significant barriers in accessing ongoing reproductive healthcare, including family planning, mental health services, and any early intervention services for her baby. She had difficulty navigating HIV prevention services and was fearful of negotiating safer sex practices with her partners.

#### 5.1.1 Risk factors

This vignette demonstrates the layers of proximal and distal, acute, and historic trauma that have an impact on many Latina immigrant women and implications for reproductive justice. Luisa had trouble in fully assessing the impact of the sexual trauma on her life for lack of counseling and other appropriate resources specializing in this needed care. This placed her at higher risk for poor mental health and negative implications for the caregiver–child relationship. The history of experiencing sexual violence, family dynamics that could fuel additional detachment, conflict, heightened levels of stress, and overall despair in Luisa are all risks for experiencing attachment/bonding difficulties with her baby. Luisa's recent arrival to the United States amid an increasingly volatile political environment contributes to added stress and anxiety that exacerbate existing barriers to reproductive and mental healthcare services. These barriers, even while living in the host country, limit the possibility of having a healthy and safe environment for both caregiver and minor children.

Luisa's story is an example of the multiple accounts by immigrant mothers who present at our clinics with their young children, in search of relief for the pain and shame caused by the unspeakable. There is evidence that the trauma of rape (Breslau et al., 1998; Cohen & Roth, 1987) can have a long‐lasting impact in women's functioning, even after years of living in the host country, affecting all areas of their life—including parental adjustment. Sexual and gender‐based violence can compromise a woman's parenting ability. In addition, the shame and fear of stigmatization and rejection by others can contribute to feelings of isolation for the caregiver.

However, not all caregivers in these circumstances develop symptoms, and not all child–parent relationships are threatened (Zraly, Rubin, & Mukamana, 2013). A caregiver's reflective capacity, sensitivity, and interactions with her child are affected by the severity of the maternal mental health symptoms, her attributions of the child (e.g., as a living reminder of her trauma), intergenerational trauma issues, the child's functioning, and the environmental support and stressors. A study conducted by Schwerdtfeger and Goff (2007) has suggested that trauma history, in general, does not negatively impact expectant mothers’ current prenatal attachment with their unborn child. However, a history of experiencing IPV and sexual abuse has been associated with poor attachment and mental health problems for both caregiver–infant and therefore needs to be identified and addressed (Cohen & Roth, 1987; Lyons‐Ruth & Block, 1996). This includes evaluating for overprotectiveness toward children (Schwerdtfeger & Goff, 2007), decreased involvement with the infant, restricted maternal affect, and disorganized attachment (Lyons‐Ruth, Bronfman, & Atwood, 1999). Our study results, as informed by this case vignette, point to the importance of understanding the role of interpersonal trauma exposure and the importance of Latina immigrant women receiving support.

### 5.2 Implications for practice: A framework for supporting caregiver–infant well‐being

There are differences in health policy between Spain and the United States, for example. Spain has a universal healthcare system, but the United States does not. However, recent policy changes in Spain have begun to potentially limit healthcare access for undocumented immigrants (Bernal‐Delgado et al., 2018). Sustaining a reproductive social justice lens to address the needs and strengths of these women requires a multipronged, multilayered approach where attention to caregiver–infant health, and therefore to the enhancement and reparation of the child–parent relationship, is central and nonnegotiable. By improving the child–parent relationship, it is possible to improve the caregiver's and the child's functioning; thus, mental health assessment and treatment with this population must include not only a relational, trauma‐informed, and developmentally based approach but also encompass a sociocultural lens (Lewis, Noroña, McConnico, & Thomas, 2013). Healthcare policy should facilitate access to quality healthcare, intervention, and services, as described next.

One of the interventions aligned with this approach is Child‐Parent Psychotherapy (CPP; Reyes, Stone, Dimmler, & Lieberman, 2017), a trauma‐focused, relationship‐based intervention for young children 0 to 6 years old and their caregivers who have experienced trauma. Its main goal is the restoration of the child–parent relationship, which is the mechanism of change. Here, the therapist's working relationship with the child–parent dyad is used as the vehicle for treatment under the premise that the quality of the relationship with the therapist will affect the child–caregiver relationship (Reyes et al., 2017). The CPP clinician serves as a translator between caregiver and child, helping the child and the parent to make meaning of trauma‐related perceptions and behaviors and to respond to each other in more realistic and positive ways (Kronenberg, 2014). All of this can restore trust and intimacy and improve ways of relating and communicating between the child and the caregiver (Lieberman & Van Horn, 2008). CPP attends to both the parent's and the child's affect regulation and physical and emotional safety, and emphasizes directly addressing and having open communication about traumatic experiences, symptoms, and reminders (Kronenberg, 2014). The model acknowledges how the past affects the present and can help families break cycles of intergenerational neglect and violence by unearthing the “ghosts” and eliciting the “angels” of the caregiver's past (Lieberman, 2007; Lieberman, Padrön, Van Horn, & Harris, 2005). The therapeutic space in CPP provides room not only to bring up painful interpersonal and collective events but also to unearth memories of safety and attunement that promote self‐worth. In CPP, clinicians are encouraged to use their reflective capacity to explore implicit biases and the impact of their own history in shaping their values and beliefs. Future research will need to explore how dyadic, relational, and culturally responsive intervention models can positively impact maternal mental health, self‐care, trauma healing, and reproductive health over time.

In general, infant mental health practitioners would best serve clients by creating safe, confidential, and culturally engaging spaces within the clinical setting for reproductive and mental health screening, inviting client‐driven/practitioner‐supported conversations which consider the culture, history, and challenges embedded in other areas of clinical presentation (Gilliam, Neustadt, & Gordon, 2009). Approaching these conversations with a spirit of “cultural humility” invites both practitioner and client to create more robust and meaningful conversations about the client's understanding of reproductive and sexual health within the context of their culture and lived experiences. Thus, discussion about options and resources relating to family planning, pregnancy, and other areas of sexual health as well as mental health services can more readily emerge and potentially be offered in a behavioral health integration model in primary care (Decker et al., 2012). Interventions have been developed which provide behavioral health consultation to primary care settings, with great potential to support caregiver and infant mental health (Byatt et al., 2016).

#### 5.2.1 Limitations

Our binary variable of exposure to physical assault is a composite from the BTQ that asks about physical assault by anyone—including friends, family members, or strangers. Thus, while IPV is included within this measure, we note the lack of a more focused measure of IPV specifically which would also include timing. Similarly, the BTQ item regarding sexual trauma does not indicate timing of the event or the perpetrator's identity. We argue that sexual or physical trauma may occur at any or all stages of migration, and that we need to conduct more research to understand the temporal association between IPV and sexual assault risk. We used a vignette, developed and based on composites from our clinical experience with Latina immigrants, to protect the confidentiality of the women we serve who are in quite vulnerable positions. Both the quantitative results of this article and the exercise of illustrating common themes can inform future research using a mixed‐methods study design including interviews with women to highlight their voices to better understand complex issues of trauma, immigration experience, maternal–child well‐being, and reproductive health. Data were not available on the actual ages of the children of the women in this study or any other details about the children themselves, thus we cannot describe differences in risk factors or health needs for women with infant children versus their counterparts with school‐age children, or the impact of risk factors on the infants. The sample is relatively small and heterogeneous regarding country of origin. The sample included in our analyses is limited to women who screened positive for mental health symptoms via the research study in primary care and who received further assessment as part of enrollment in the treatment study. Results are therefore not generalizable to community samples of women or to those who do not access any healthcare or community organization supports. We do not have further data for women who screened negative. Further research on the impact on mothers of variations in healthcare policy for immigrant women is needed.

### 5.3 Conclusions: Healthcare practice and policy implications for immigrant women

Political legislation in both the United States and Spain have only further potentially limited access to services for immigrant populations since this study was conducted. Redefining women's access to quality and holistic healthcare that considers the transnational experiences and sociocultural–political–historical legacies affecting pregnant and parenting immigrant women while reducing disparities in maternal and child health is a critical public health goal. We recommend the following considerations:

- Screening Latina immigrant women and all mothers for depression and stress in maternal and child well visits and universally in primary care. This is, in fact, an important and growing practice in working with all mothers and young children in primary care and in combination with the assessment of infant development and mental health.
- Training for clinicians and systems of care in addressing trauma using dyadic maternal–child therapies such as CPP, which provides a culturally responsive and attachment supporting intervention (Lieberman & Van Horn, 2008).
- Eliminating health care and immigration policies that create barriers for immigrant women's access to reproductive, mental health and other healthcare.
- Encompass a sociocultural lens (Lewis et al., 2013) for an exploration of how trauma and sociopolitical context have impacted the caregivers’ and family's identity and functioning as well as the child's socioemotional development.
- Facilitate engagement through a collaborative process with the caregiver(s) regarding scope and goals of intervention and for the identification of their perceived resources, needs, values, and meaning‐making of inequities and injustices (Lewis et al., 2013, p. 17).
- Be aware of the implicit biases of our professional and organizational cultures.

Otherwise, we risk becoming bystanders when systems fail to address the needs of immigrant Latin American women. Change and reflection starts with the self; however, this cannot be done in isolation. Reflective practice/supervision have been identified as effective strategies in promoting self‐reflection, changing practice, and preventing secondary traumatic stress in the work with young families affected by trauma. These recommendations, from a reproductive justice lens, have the potential to change the trajectory and intergenerational consequences of trauma in the lives of women and children.

## CONFLICT OF INTEREST

The authors report no conflicts of interest.

## Supporting information

SUPPORTING INFORMATION

## APPENDIX 1

### 1.1

**Table A1:** Sample descriptive summary by maternal status (*N* = 154)

	Women with no minor children (*n* = 98)		Women with minor children (*n* = 56)		
	*n*	%	*n*	%	*P*‐value
Age category					
18–34	59	60.2	20	35.7	0.000
35–49	17	17.3	32	57.1	
50+	22	22.4	4	7.1	
U.S./Spain citizenship					
Noncitizen	90	91.8	47	83.9	0.132
Citizen	8	8.2	9	16.1	
Race					
White	19	19.4	8	14.3	0.044
Black	5	5.1	2	3.6	
Indigenous/Native American	5	5.1	9	16.1	
Hispanic/Latino/Caribbean	3	3.1	6	10.7	
Mixed	66	67.3	31	55.4	
Education Level					
<High school	23	23.5	29	51.8	0.000
High‐school diploma, GED, vocational school, or higher	75	76.5	27	48.2	
Last year sexual partner status					
Only have main partner	42	42.9	29	51.8	0.512
Only have casual partner	9	9.2	7	12.5	
Both have main partner and casual partner	24	24.5	11	19.6	
Don't have main partner or casual partner	23	23.5	9	16.1	
No. of children					
<3	98	100.0	51	91.1	0.003
≥3		0.0	5	8.9	
Physical assault					
No	44	44.9	16	28.6	0.046
Yes	54	55.1	40	71.4	
Sexual Trauma					
No	71	72.4	39	69.6	0.711
Yes	27	27.6	17	30.4	
Last year sexual behavior status					
Unprotected sexual behavior	50	51.0	34	60.7	0.403
Protected sexual behavior	24	24.5	13	23.2	
No sexual behavior	24	24.5	9	16.1	
Met criteria for PTSD (PCL‐5 33+)	47	48.0	22	39.3	0.298
	*M*	*SD*	*M*	*SD*	*P*‐value
Depression (PHQ‐9) (0–27)	12.5	5.9	13.5	5.4	0.286
Generalized anxiety (GAD‐7) (0–21)	9.4	4.7	10.9	5.2	0.085
PTSD (PCL) (0–80)	30.2	17.0	29.7	17.4	0.853
Alcohol abuse (AUDIT) (0–12)	4.6	3.1	4.3	3.3	0.623
Drug abuse (DAST) (0–9)	1.0	1.9	0.9	1.6	0.752
Benzodiazepines (0–20)	3.4	5.1	3.2	4.9	0.753
*M* Age of immigration	25.77	10.55	24.82	8.17	0.565
Length of time in resident country	9.01	8.69	12.16	6.73	0.021

*Note*. GED = general equivalency diploma; PTSD = posttraumatic stress disorder; PCL‐5 = Post‐Traumatic Stress Disorder Checklist, Civilian version; PHQ‐9 = Patient Health Questionnaire (9‐item); GAD‐7 = General Anxiety Disorder screener; AUDIT = Alcohol Use Disorders Identification Test; DAST = Drug Abuse Screening Test.

## References

[imhj21805-bib-0001] Agency for Healthcare Research and Quality . (2015). 2014 National healthcare quality and disparities report. AHRQ Publication No. 15‐0007. Rockville, MD: Agency for Healthcare Research and Quality.

[imhj21805-bib-0002] Alegría, M. , Álvarez, K. , & DiMarzio, K. (2017). Immigration and mental health. Current Epidemiology Reports, 4(2), 145–155. 10.1007/s40471-017-0111-2 29805955 PMC5966037

[imhj21805-bib-0003] Alegría, M. , Falgas Bague, I. , Collazos, F. , Carmona Camacho, R. , Lapatin Markle, S. , Wang, Y. , … Shrout, P. E. (2019). Evaluation of the integrated intervention for dual problems and early action among Latino immigrants with co‐occurring mental health and substance misuse symptoms: A randomized clinical trial. JAMA Network Open, 2(1), e186927–e186927 10.1001/jamanetworkopen.2018.6927 30646205 PMC6484537

[imhj21805-bib-0004] Alegría, M. , NeMoyer, A. , Falgas Bague, I. , Wang, Y. , & Alvarez, K. (2018). Social determinants of mental health: Where we are and where we need to go. Current Psychiatry Reports, 20(11), 95 10.1007/s11920-018-0969-9 30221308 PMC6181118

[imhj21805-bib-0005] Alegría, M. , Takeuchi, D. , Canino, G. , Duan, N. , Shrout, P. , Meng, X.‐L. , … Gong, F. (2004). Considering context, place and culture: The National Latino and Asian American Study. International Journal of Methods in Psychiatric Research, 13(4), 208–220. 10.1002/mpr.178 15719529 PMC2774128

[imhj21805-bib-0006] Alegría, M. , Vila, D. , Woo, M. , Canino, G. , Takeuchi, D. , Vera, M. , … Shrout, P. (2004). Cultural relevance and equivalence in the NLAAS instrument: Integrating etic and emic in the development of cross‐cultural measures for a psychiatric epidemiology and services study of Latinos. International Journal of Methods in Psychiatric Research, 13(4), 270–288. https://doi-org.ezproxy.bu.edu/10.1002/mpr.181 15719532 10.1002/mpr.181 PMC2771729

[imhj21805-bib-0007] American Psychiatric Association . (2013). Diagnostic and statistical manual of mental disorders (DSM‐5). Arlington, VA: Author.

[imhj21805-bib-0008] Arias, I. (2004). The legacy of child maltreatment: Long‐term health consequences for women. Journal of Women's Health, 13(5), 468–473. 10.1089/1540999041280990 15257839

[imhj21805-bib-0009] Babor, T. F. , Higgins‐Biddle, J. C. , Saunders, J. B. , & Monteiro, M. G. (2001). Audit. The Alcohol Use Disorders Identification Test (AUDIT): Guidelines for use in primary care. Retrieved from https://apps.who.int/iris/handle/10665/67205

[imhj21805-bib-0010] Balbernie, R. (2002). An infant in context: Multiple risks, and a relationship. Infant Mental Health Journal, 23(3), 329–341. 10.1002/imhj.10019

[imhj21805-bib-0011] Batsaikhan, U. , Darvas, Z. , & Raposo, I. G. (2018). People on the move: Migration and mobility in the European Union. Brussels, Belgium: Bruegel.org. Retrieved from http://bruegel.org/wp-content/uploads/2018/01/People_on_the_move_ONLINE.pdf

[imhj21805-bib-0012] Bernal‐Delgado, E. , García‐Armesto, S. , Oliva, J. , Sánchez Martínez, F. I. , Repullo, J. R. , Peña‐Longobardo , … Hernández‐Quevedo, C. (2018). Spain: Health system review. Health Systems in Transition, 20(2), 1–179. http://www.euro.who.int/__data/assets/pdf_file/0008/378620/hit-spain-eng.pdf?ua=1 30277216

[imhj21805-bib-0013] Blanchard, E. B. , Jones‐Alexander, J. , Buckley, T. C. , & Forneris, C. A. (1996). Psychometric properties of the PTSD Checklist (PCL). Behaviour Research and Therapy, 34(8), 669–673. 10.1016/0005-7967(96)00033-2 8870294

[imhj21805-bib-0014] Breslau, N. , Kessler, R. C. , Chilcoat, H. D. , Schultz, L. R. , Davis, G. C. , & Andreski, P. (1998). Trauma and posttraumatic stress disorder in the community: The 1996 Detroit Area Survey of Trauma. Archives of General Psychiatry, 55(7), 626–632. 10.1001/archpsyc.55.7.626 9672053

[imhj21805-bib-0015] Bronfenbrenner, U. (1979). The ecology of human development: Experiments by nature and design. Cambridge, MA: Harvard University Press.

[imhj21805-bib-0016] Byatt, N. , Biebel, K. , Moore Simas, T. A. , Sarvet, B. , Ravech, M. , Allison, J. , & Straus, J. (2016). Improving perinatal depression care: The Massachusetts Child Psychiatry Access Project for Moms. General Hospital Psychiatry, 40, 12–17. 10.1016/j.genhosppsych.2016.03.002 27079616

[imhj21805-bib-0017] Cohen, L. J. , & Roth, S. (1987). The psychological aftermath of rape: Long‐term effects and individual differences in recovery. Journal of Social and Clinical Psychology, 5(4), 525–534. 10.1521/jscp.1987.5.4.525

[imhj21805-bib-0018] Collazos, F. , Markle, S. L. , Chavez, L. , Brugal, M. T. , Aroca, P. , Wang, Y. , … Alegría, M. (2019). HIV testing in clinical and community settings for an international sample of Latino immigrants and nonimmigrants. Journal of Latinx Psychology, 7(1), 59–75. 10.1037/lat0000101 30859017 PMC6407700

[imhj21805-bib-0019] Copelon, R. , & Petchesky, R. (1995). Reproductive and sexual rights as human rights In Shuler M. (Ed.), Basic needs to basic rights (pp. 343–367). Washington, DC: Women, Law and Development International.

[imhj21805-bib-0020] Coutinho, E. , Almeida, F. , Duarte, J. , Chaves, C. , Nelas, P. , & Amaral, O. (2015). Factors related to domestic violence in pregnant women. Procedia ‐ Social and Behavioral Sciences, 171, 1280–1287. 10.1016/j.sbspro.2015.01.242

[imhj21805-bib-0021] Crenshaw, K. (2005). Mapping the margins: Intersectionality, identity politics, and violence against women of color (1994) In Bergen R. K., Edleson J. L., & Renzetti C. M. (Eds.), Violence against women: Classic papers (pp. 282–313). Auckland, New Zealand: Pearson Education New Zealand.

[imhj21805-bib-0022] Decker, M. R. , Frattaroli, S. , McCaw, B. , Coker, A. L. , Miller, E. , Sharps, P. , … Gielen, A. (2012). Transforming the healthcare response to intimate partner violence and taking best practices to scale. Journal of Women's Health, 21(12), 1222–1229. 10.1089/jwh.2012.4058 PMC3654819 23210490

[imhj21805-bib-0023] Denov, M. (2015). Children born of wartime rape: The intergenerational realities of sexual violence and abuse. Ethics, Medicine & Public Health, 1(1), 61–68. 10.1016/j.jemep.2015.02.001

[imhj21805-bib-0024] Dezetter, A. , Briffault, X. , Bruffaerts, R. , De Graaf, R. , Alonso, J. , Konig, H. H. , … Kovess‐Masfety, V. (2013). Use of general practitioners versus mental health professionals in six European countries: The decisive role of the organization of mental health‐care systems. Social Psychiatry and Psychiatric Epidemiology, 48(1), 137–149. 10.1007/s00127-012-0522-9 22644000

[imhj21805-bib-0025] Falgas, I. , Ramos, Z. , Herrera, L. , Qureshi, A. , Chavez, L. , Bonal, C. , … Alegría, M. (2017). Barriers to and correlates of retention in behavioral health treatment among Latinos in 2 different host countries: The United States and Spain. Journal of Public Health Management & Practice, 23(1), e20–e27. 10.1097/PHH.0000000000000391 26910867 PMC5320890

[imhj21805-bib-0026] Fortuna, L. R. , Porche, M. V. , & Alegría, M. (2008). Political violence, psychosocial trauma, and the context of mental health services use among immigrant Latinos in the United States. Ethnicity & Health, 13(5), 435–463. 10.1080/13557850701837286 18850369 PMC2771411

[imhj21805-bib-0027] Fortuna, L. R. , Ramos, Z. , Fuentes, L. , Falgas, I. , & Alegría, M. (2017 March). Development of the IIDEA Therapy for Latino immigrant adults with co‐occuring disorders. Paper presented at the V International Congress of Dual Disorders, Madrid, Spain.

[imhj21805-bib-0028] Galano, M. M. , Grogan‐Kaylor, A. C. , Stein, S. F. , Clark, H. M. , & Graham‐Bermann, S. A. (2016). Posttraumatic stress disorder in Latina women: Examining the efficacy of the Moms’ Empowerment Program. Psychological Trauma: Theory, Research, Practice, and Policy, 9(3), 344–351. 10.1037/tra0000218 27869463

[imhj21805-bib-0029] Gilliam, M. L. , Neustadt, A. , & Gordon, R. (2009). A call to incorporate a reproductive justice agenda into reproductive health clinical practice and policy. Contraception, 79(4), 243–246. 10.1016/j.contraception.2008.12.004 19272493

[imhj21805-bib-0030] Gómez, A. M. , & Speizer, I. S. (2009). Intersections between childhood abuse and adult intimate partner violence among Ecuadorian women. Maternal and Child Health Journal, 13(4), 559–566. 10.1007/s10995-008-0387-4 18649129

[imhj21805-bib-0031] Gourlay, A. , Mshana, G. , Birdthistle, I. , Bulugu, G. , Zaba, B. , & Urassa, M. (2014). Using vignettes in qualitative research to explore barriers and facilitating factors to the uptake of prevention of mother‐to‐child transmission services in rural Tanzania: A critical analysis. BMC Medical Research Methodology, 14(1), 21. 10.1186/1471-2288-14-21 PMC3922981 24512206

[imhj21805-bib-1003] Huang, F. Y. , Chung, H. , Kroenke, K. , Delucchi, K. L. , & Spitzer, R. L. (2006). Using the patient health questionnaire‐9 to measure depression among racially and ethnically diverse primary care patients. Journal of General Internal Medicine, 21(6), 547–552. 10.1111/j.1525-1497.2006.00409.x 16808734 PMC1924626

[imhj21805-bib-0032] Kaltman, S. , Green, B. L. , Mete, M. , Shara, N. , & Miranda, J. (2010). Trauma, depression, and comorbid PTSD/depression in a community sample of Latina immigrants. Psychological Trauma: Theory, Research, Practice, and Policy, 2(1), 31–39. https://doi-org/ezproxy.bu.edu/10.1037/a0018952 10.1037/a0018952 PMC2850073 20376305

[imhj21805-bib-0033] Kroenke, K. , Spitzer, R. L. , & Williams, J. B. W. (2001). The PHQ‐9: Validity of a brief depression severity measure. Journal of General Internal Medicine, 16(9), 606–613. 10.1046/j.1525-1497.2001.016009606.x 11556941 PMC1495268

[imhj21805-bib-0034] Kronenberg, M. (2014). Child‐parent psychotherapy: An overview In Allen B. & Kronenberg M. (Eds.), Treating traumatized children: A casebook of evidence‐based therapies (pp. 103–120). New York, NY: Guilford Press.

[imhj21805-bib-0035] Lewis, M. l. , Noroña, C. R. , McConnico, N. , & Thomas, K. (2013). Colorism, a legacy of historical trauma in parent‐child relationships: Clinical, research and personal perspectives. ZERO TO THREE Journal, 34(2), 11–23.

[imhj21805-bib-0036] Lieberman, A. F. (2007). Ghosts and angels: Intergenerational patterns in the transmission and treatment of the traumatic sequelae of domestic violence. Infant Mental Health Journal, 28(4), 422–439. 10.1002/imhj.20145 28640404

[imhj21805-bib-0037] Lieberman, A. F. , Padrön, E. , Van Horn, P. , & Harris, W. W. (2005). Angels in the nursery: The intergenerational transmission of benevolent parental influences. Infant Mental Health Journal, 26(6), 504–520. 10.1002/imhj.20071 28682485

[imhj21805-bib-0038] Lieberman, A. F. , & Van Horn, P. (1998). Attachment, trauma, and domestic violence: Implications for child custody. Child & Adolescent Psychiatric Clinics of North America, 7(2), 423–443. https://doi-org.ezproxy.bu.edu/10.1016/S1056-4993(18)30250-5 9894073

[imhj21805-bib-0039] Lieberman, A. F. , & Van Horn, P. (2008). Psychotherapy with infants and young children: Repairing the effects of stress and trauma on early attachment. New York, NY: Guilford Press.

[imhj21805-bib-0040] Lieberman, A. F. , Van Horn, P. , & Ippen, C. G. (2005). Toward evidence‐based treatment: Child‐parent psychotherapy with preschoolers exposed to marital violence. Journal of the American Academy of Child & Adolescent Psychiatry, 44(12), 1241–1248. 10.1097/01.chi.0000181047.59702.58 16292115

[imhj21805-bib-0041] Lyons‐Ruth, K. , & Block, D. (1996). The disturbed caregiving system: Relations among childhood trauma, maternal caregiving, and infant affect and attachment. Infant Mental Health Journal, 17(3), 257–275. 10.1002/(SICI)1097-0355(199623)17:3

[imhj21805-bib-0042] Lyons‐Ruth, K. , Bronfman, E. , & Atwood, G. (1999). A relational diathesis model of hostile‐helpless states of mind: Expressions in mother–infant interaction In Solomon J. & George C. (Eds.), Attachment disorganization (pp. 33–70). New York, NY: Guilford Press.

[imhj21805-bib-0043] Minaya, O. , Fresan, A. , Cortes‐Lopez, J. L. , Nanni, R. , & Ugalde, O. (2011). The Benzodiazepine Dependence Questionnaire (BDEPQ): Validity and reliability in Mexican psychiatric patients. Addictive Behaviors, 36(8), 874–877. 10.1016/j.addbeh.2011.03.007 21481543

[imhj21805-bib-0044] Nash, E. , Gold, R. B. , Ansari‐Thomas, Z. , Cappello, O. , & Mohammed, L. (2017). Policy trends in the States: 2016. Retrieved from https://www.guttmacher.org/article/2017/01/policy-trends-states-2016

[imhj21805-bib-0045] National Latina Institute for Reproductive Health . (n.d.). Health care access. Retrieved from http://www.latinainstitute.org/en/what-we-do/healthcare-access

[imhj21805-bib-0046] National Women's Law Center . (2017). Immigrant rights and reproductive justice: How harsh immigration policies harm immigrant health. Retrieved from https://nwlc.org/wp-content/uploads/2017/04/Immigrant-Rights-and-Reproductive-Justice.pdf

[imhj21805-bib-0047] Ogbonnaya, I. N. , Finno‐Velasquez, M. , & Kohl, P. L. (2015). Domestic violence and immigration status among Latina mothers in the child welfare system: Findings from the National Survey of Child and Adolescent Well‐Being II (NSCAW II). Child Abuse & Neglect, 39, 197–206. 10.1016/j.chiabu.2014.10.009 25459990

[imhj21805-bib-1001] Patient Protection and Affordable Care Act (2010). 42 U.S.C. § 18001.

[imhj21805-bib-0048] Phipps, R. M. , & Degges‐White, S. (2014). A new look at transgenerational trauma transmission: Second‐generation Latino immigrant youth. Journal of Multicultural Counseling and Development, 42(3), 174–187. https://doi-org.ezproxy.bu.edu/10.1002/j.2161-1912.2014.00053.x

[imhj21805-bib-0049] Ramos, Z. , Fortuna, L. R. , Porche, M. V. , Wang, Y. , Shrout, P. E. , Loder, S. , … Alegría, M. (2017). Posttraumatic stress symptoms and their relationship to drug and alcohol use in an international sample of Latino immigrants. Journal of Immigrant and Minority Health, 19(3), 552–561. 10.1007/s10903-016-0426-y 27150593 PMC5322239

[imhj21805-bib-0050] Reyes, V. , Stone, B. J. , Dimmler, M. H. , & Lieberman, A. F. (2017). Child‐parent psychotherapy: An evidence‐based treatment for infants and young children In Landolt M. A., Cloitre M., & Schnyder U. (Eds.), Evidence‐based treatments for trauma related disorders in children and adolescents (pp. 321–340). Cham, Switzerland: Springer.

[imhj21805-bib-0051] Ross, L. , & Solinger, R. (2017). Reproductive justice: An introduction. Oakland, CA: University of California Press.

[imhj21805-bib-0052] Saloner, B. , Bandara, S. , Bachhuber, M. , & Barry, C. L. (2017). Insurance coverage and treatment use under the Affordable Care Act among adults with mental and substance use disorders. Psychiatric Services, 68(6), 542–548. 10.1176/appi.ps.201600182 28093059

[imhj21805-bib-0053] Schnurr, P. , Vielhauer, M. , Weathers, F. , & Findler, M. (1999). The brief trauma questionnaire. Retrieved from https://www.ptsd.va.gov/professional/assessment/documents/BTQ.pdf

[imhj21805-bib-1002] Schore, A. N. (2013). Relational trauma, brain development, and dissociation In Ford J. D. & Courtois C. A. (Eds.), Treating complex traumatic stress disorders in children and adolescents: Scientific foundations and therapeutic models (pp. 3–23). New York, NY: The Guilford Press.

[imhj21805-bib-0054] Schwerdtfeger, K. L. , & Goff, B. S. N. (2007). Intergenerational transmission of trauma: Exploring mother–Infant prenatal attachment. Journal of Traumatic Stress, 20(1), 39–51. 10.1002/jts.20179 17345647

[imhj21805-bib-0055] Skinner, H. A. (1982). The Drug Abuse Screening Test. Addictive Behaviors, 7(4), 363–371. 7183189 10.1016/0306-4603(82)90005-3

[imhj21805-bib-0056] Smith, A. (2005). Beyond pro‐choice versus pro‐life: Women of color and reproductive justice. NWSA Journal, 17(1), 119–140.

[imhj21805-bib-0057] Spitzer, R. L. , Kroenke, K. , Williams, J. B. W. , & Löwe, B. (2006). A brief measure for assessing generalized anxiety disorder: The GAD‐7. Archives of Internal Medicine, 166(10), 1092–1097. 10.1001/archinte.166.10.1092 16717171

[imhj21805-bib-0058] StataCorp . (2015). Stata: Release 14 [Computer Software]. College Station, TX: Author.

[imhj21805-bib-0059] Vega, W. A. , Kolody, B. , Valle, J. R. , & Hough, R. (1986). Depressive symptoms and their correlates among immigrant Mexican women in the United States. Social Science & Medicine, 22(6), 645–652. 10.1016/0277-9536(86)90037-7 3715504

[imhj21805-bib-0060] Zraly, M. , Rubin, S. E. , & Mukamana, D. (2013). Motherhood and resilience among Rwandan genocide‐rape survivors. Ethos, 41(4), 411–439. 10.1111/etho.12031

