# Supplementary material for: Trauma, immigration, and sexual health among Latina women: Implications for maternal–child well‐being and reproductive justice
Source: Infant Ment Health J. 2019 Jul 23;40(5):640–58. doi: 10.1002/imhj.21805 (PMC6972560; doi:10.1002/imhj.21805)
Supplement: Supplementary file 1 — SUPPORTING INFORMATION [file IMHJ-40-640-s001.docx]

**Supplemental Material**

**Illustrative Vignette #2**

Graciela is a 34-year-old mother who has been married to her current partner, and father of their newborn baby, for two years. Her previous marriage ended in divorce as a result of her first husband being unfaithful and emotionally and physically violent. Graciela and this man had two children in their home country of Honduras, and within a very poor community. Due to the difficulties (danger, lack of financial resources) of migrating with her two children she left them with her sister and brother-in-law when she made the difficult decision to leave the increasingly abusive marital relationship. Upon unauthorized entry to the U.S. she resided with a cousin, found work, and sent financial support back home for her children. After eight years of separation from her family, with only regular phone contact with her children (ages 9 and 10), she has felt overwhelmingly guilty and sad after the birth of her new baby. She reports difficulty bonding with the baby, but also that she receives emotional support from her new husband and the few extended family members who reside in the region.

Medical history reveals that Graciela had a difficult pregnancy and, because of her documentation status and other economic, social, and language factors, she was not able to stabilize the various medical challenges at different points of the pregnancy that ultimately resulted in an emergency C-section. Graciela expressed concern that she was somehow being “punished by God” for leaving her children behind and that the fate of this pregnancy was out of her hands. At one point of her pregnancy, she was evaluated for acute depression but due to her status, was not able to access ongoing counseling. Graciela’s medical team, while attentive, was not trained in working with the immigrant community and there were no Spanish speaking staff at the clinic or hospital where she received her care. Graciela was frequently seen reading her Bible during her stays in the hospital or while waiting in the clinic.

Graciela has become increasingly anxious and worried for the well-being of her children in Honduras, as the stories of community and political violence there have increased, with her older daughter receiving threats from gang members in her local pueblo back home. Graciela has become preoccupied with finding a way to reunite with her children, which has further distracted her from bonding and attachment with her new baby. Her husband, while attentive and supportive, works 60-70 hours a week to support his new family, especially since Graciela will not be able to work for several weeks.

Graciela’s story offers insights into the circumstances that an older immigrant mother with a new baby can face, including being preoccupied for the well-being of children and family in two countries. For many mothers who left their children behind in their country of origin a new pregnancy resurfaces the unresolved pain caused by this type of silent and ambiguous grief. The tendency to pathologize Graciela’s symptoms by clinical professionals at the expense of contextualizing her challenges more holistically and through an intergenerational, socio-cultural, and transnational lens presents an example of a clear but important challenge to medical and mental health care providers who are often constrained by the limitations of their training. Current literature highlights how obstetric and gynecological clinician education on reproductive justice can inform providers of how immigration, discrimination, and socioeconomic status are connected to low rates of prenatal and late entry care, delays in seeking abortion, high rates of STD and HIV, and higher rates of unintended pregnancies. Thus, clinicians can tailor their out-reach services and professionally advocate for policies and practices to reduce barriers to accessing contraception, infertility treatment, general reproductive care, abortion services, and screen for sexual violence (Gilliam, Neustadt, & Gordon, 2009).

Clinical attention is on the mother’s depression, with little mention about the baby and his relationship to this mother and the larger family system. In fact, the baby contributes to the mother’s capacity to love and bond despite her struggles. From a transactional perspective, the baby’s strengths and the mother-baby moments of quality attunement can be used as ports of entry and scaffolding to engage the mother. This engagement can increase the mother’s sense of competence that is otherwise threatened by the shadow of loss and the heightened sense of stress. Additionally, this can empower the mother and provide hope about her protective abilities despite all odds. The political environment on immigration likely imposes additional stress and anxiety on a fragile family system, and situations like that of Graciela demonstrate once again, the proximal, the distal, the historical, and interpersonal factors at play on all issues of mental health, reproductive health, and maternal-child well-being.
